# Are People More Likely to Vape or Smoke Indoors? A Population Survey of Adults in England

**DOI:** 10.1093/ntr/ntae094

**Published:** 2024-04-18

**Authors:** Harry Tattan-Birch, Sarah E Jackson, Lion Shahab, Jamie Brown

**Affiliations:** Department of Behavioural Science and Health, University College London, London, UK; SPECTRUM Consortium, UK; Department of Behavioural Science and Health, University College London, London, UK; SPECTRUM Consortium, UK; Department of Behavioural Science and Health, University College London, London, UK; SPECTRUM Consortium, UK; Department of Behavioural Science and Health, University College London, London, UK; SPECTRUM Consortium, UK

## Abstract

**Background:**

Increasingly, people smoke cigarettes outdoors and avoid exposing bystanders to harm. People may not have the same motivation to vape outdoors since e-cigarettes, unlike cigarettes, do not create side stream emissions and exhaled aerosol contains fewer toxicants than secondhand smoke. This study aims to estimate the prevalence and correlates of vaping and smoking indoors among adults in England.

**Aims and Methods:**

Data came from the Health Survey for England 2019, a cross-sectional household survey. Adults who vape or smoke (*N* = 1530) were asked whether they had vaped or smoked indoors inside the home, car, or other places within the past 7 days. Logistic regression was used to estimate prevalence and key correlates of indoor use, including age, sex, presence of adults/children in home, housing tenure, and nicotine dependence.

**Results:**

People who exclusively vaped were much more likely to use their product indoors than those who exclusively smoked (87.0% vs. 52.0%; odds ratio [OR] = 6.16, 95% confidence interval [CI] = 4.09 to 9.28). Similarly, people who dual used had higher odds of vaping than smoking indoors (62.1% vs. 44.3%; OR = 3.76, 95% CI = 2.06 to 6.84). The preference for vaping over smoking indoors was found across different locations, including at home and in cars. Those who were older, highly dependent on nicotine, and lived alone were most prone to use any product indoors. While housing tenure was not strongly associated with vaping indoors, those living in social housing were much more likely to smoke indoors than homeowners.

**Conclusions:**

Adults in England are much more likely to vape than smoke indoors. Age, nicotine dependence, and living alone are strongly associated with both behaviors.

**Implications:**

Our results show that people have a strong preference for vaping over smoking indoors, including in the home. Given the high prevalence of vaping indoors, policy makers, landlords, and businesses must weigh up the ethics, benefits, and harms of extending smoke-free laws to include e-cigarettes.

## Background

Since the 1980s, there has been rising awareness about the harmful effects of secondhand tobacco smoking (ie, exposure to other people’s tobacco smoke) on health. Most of the harm comes from exposure to smoking cigarettes in enclosed spaces such as indoors in homes, workplaces, bars, and cars.^1^ These concerns led to a shift in social norms in many countries, such that people increasingly avoid smoking indoors, where the risks of secondhand smoke are most pronounced.^2–4^ For instance, in English homes, a growing proportion of parents are choosing to only smoke outdoors, which has resulted in a 90% reduction in children’s intake of secondhand smoke over the past two decades.^5^ Many countries have also introduced legislation that bans smoking indoors in cars and public places such as restaurants, bars, and workplaces.^6–9^ Recently, focus has been placed on the growth in e-cigarette use (“vaping”; https://addictovocab.org/ADDICTO:0000212), which poses both opportunities and threats for secondhand exposure.

On the one hand, e-cigarette aerosol contains much lower levels of toxicants and carcinogens than cigarette smoke^10–13^ and, unlike cigarettes which release side stream smoke, the user retains the vast majority of aerosol produced while vaping (median retention of 99.6% for nicotine, 94.8% for glycerin, and 98.3% for propylene glycol).^14,15^ Thus, secondhand vaping is likely to be much less hazardous to the health of bystanders than secondhand smoking. This means that if people vape indoors as a way to avoid smoking indoors, this likely reduces the harm caused to bystanders. On the other hand, secondhand vaping is not entirely harmless.^16^ For instance, as with cigarette smoke, there are potential dangers to people with allergies to specific flavorings or chemicals.^17^ If people feel more comfortable vaping than smoking indoors, then rising vaping prevalence would expose more bystanders to these risks. Moreover, even if secondhand vaping were completely harmless to the health bystanders, there are concerns that it might “renormalize” the use of nicotine indoors, possibly encouraging (i) more smokers to light up indoors or (ii) people who have never used nicotine to start vaping or smoking themselves.^18–20^ Counter to this, it is possible that vaping could further denormalize secondhand smoking by providing people with an alternative to smoking indoors.^21,22^

There are several reasons why people may be more comfortable vaping than smoking indoors. Firstly, e-cigarette aerosol is much less toxic than cigarette smoke, as discussed above.^10–13^ Therefore, concerns about harming others—including one’s own children—is likely a stronger motive for people to take their smoking rather than vaping outdoors, and bystanders may also be more willing to accept exposure to the latter than the former. Second, smoking is much harder to conceal than vaping, because of the odor produced by burning tobacco and need to consume a full cigarette while it is alight (rather than taking a couple of puffs).^23^ Third, related to the previous point, the smell of e-cigarette aerosol may be less aversive to bystanders than cigarette smoke, and it does not linger in buildings and on furniture to the same extent, if at all.^24^ Finally, many private landlords have rules against smoking indoors in the home, and some countries (including the UK, Canada, France, and Bahrain) have banned smoking in the car when young passengers are present, and others have smokefree rules for social housing.^6–9,25^ Often, these laws do not extend to e-cigarettes, allowing people to vape openly in places where they would not be allowed to smoke (although there are calls to include e-cigarettes in these bans).^26,27^ Even in places where there are rules against vaping indoors, smoke alarms usually do not have the sensitivity to detect exhaled e-cigarette aerosol, allowing people to “stealth vape.”^23^

Demographic characteristics and socio-economic circumstances may explain why some people smoke or vape indoors while others do not. People who live with children are more motivated to take their smoking outdoors than those who live alone or with other adults,^28^ though it is unclear whether the same is true for vaping. Those living in social housing and multiunit buildings, often without a private garden, are more likely to smoke indoors than those in owner-occupied homes, but again it is unclear if this is also the case for vaping.^29^ People who are more dependent on nicotine, including those who smoke or vape shortly after waking up in the morning,^30^ might find it more difficult to resist urges to use their product indoors.

Using data from the Health Survey for England 2019, we aim to estimate, among people who exclusively smoke, exclusively vape, and dual use, the percentage that report having recently used their nicotine products indoors (anywhere indoors, indoors at home, in the car, and/or other indoor places). In exclusive users, we also aimed to examine how the prevalence of indoor vaping and smoking varies by age, sex, housing tenure, presence of a child or adult in the home, smoking and vaping history, and nicotine dependence.

## Methods

### Design

Data came from the Health Survey for England (HSE) in 2019 (January–December), a cross-sectional survey that used a stratified, clustered, multistage design to recruit a representative sample of homes in England. In the homes that were selected, every adult was offered an interview. Of the eligible homes, 60% participated, and 84% of adults in these households were interviewed. Non-response was accounted for using survey weights, as described in detail in the technical appendix (available online).

### Participants

Adults (aged ≥16) were eligible for inclusion in this study if they reported current vaping or smoking (see Measures below).

### Measures

#### Demographics and Potential Correlates

Several demographic variables were reported for all members of a given home from a single individual (the household reference person). These included sex (male/female), age, the presence of children or other adults (living with children/only adults/alone), and housing tenure (owned/privately-rented/social housing). Age was provided in 2- or 5-year age bands (exact age imputed as the middle age of the relevant band). Housing tenure was ascertained by asking whether the home was rented or owner-occupied (with or without a mortgage). Renters were asked, “Who is your landlord?.” Homes where the landlord was “the local authority/council” or “a housing association or cooperative or charitable trust or registered social landlord” were labeled as social housing, while all others were considered privately-rented.

Participants were asked, “Do you smoke cigarettes at all nowadays?.” Those who responded “Yes” were considered to smoke currently. Similarly, people were classified as currently vaping if they responded “Yes” to the question, “Do you use an e-cigarette or vaping device at all nowadays?.” Dual use was indicated by responding “Yes” to both questions, exclusive smoking by responding “Yes” to smoking but not vaping, and vice versa for exclusive vaping.

Past regular smoking history was ascertained in people who exclusively vape, and past regular vaping history in people who exclusively smoke. People who said they, “Smoked cigarettes regularly, at least 1 per day” were classified as having previously smoked regularly (vs. not). People were asked whether they had ever tried an e-cigarette. Those who responded “Yes” were considered to have previously vaped regularly, while those who responded “No” or “Yes, but only once or twice” were considered to not have done so.

Nicotine dependence was operationalized as time to first smoke/vape after waking up, with non-daily use added as the least dependent category. Time to first cigarette after waking up has been consistently validated as one of the most important markers of cigarette dependence, being highly predictive of whether a person will relapse after they attempt to quit.^30^ Moreover, unlike many other markers of cigarette dependence (eg, cigarettes per day), it can be directly applied to e-cigarette dependence. Non-daily use was defined as reporting not smoking cigarettes “every day or almost every day” or not vaping “every day” in the past month. People who smoked daily were asked, “How soon after waking do you usually smoke your first cigarette of the day?,” while those who vaped daily were asked, “How soon after waking do you usually have your first e-cigarette or vape of the day?.” Responses were coded into a seven-point scale of nicotine dependence:

1. Non-daily
2. Daily, use within “2 h or more”
3. Daily, use within “1 h but <2 h”
4. Daily, use within “30 min but <1 h”
5. Daily, use within “15–29 min”
6. Daily, use within “5–14 min”
7. Daily, use within “<5 min”

People who dual used had a separate score for both e-cigarettes and cigarettes, so were assigned a nicotine dependence score equal to whichever was higher. For example, someone who vaped less than daily but smoked every day within 5 min of waking would be classified as highly dependent (ie, a score of 7).

### Outcomes

People who reported smoking were asked, “In which of these places, if any, did you smoke in during the last 7 days ending yesterday?.” Responses were categorized as follows:

1. Indoors at home: “At my home, indoors”
2. Inside car: “While traveling by car”
3. Other indoor places: “Inside other people’s homes” or “Inside other places”

People who reported any of 1–3 were considered to have smoked indoors, while those who reported none of the above were considered to not have smoked indoors. Analogous questions about vaping indoors were asked to vapers and classified equivalently.

### Analysis

As pre-specified, we performed the analysis on complete cases because fewer than 5% of participants had missing data on any of the outcomes or covariates. Sample characteristics were reported overall and stratified into groups who exclusively smoke, exclusively vape, or dual use.

Categorical outcomes were analyzed using binary or multinomial logistic regression, accounting for non-response weights and clustering using the *survey* package in *R*.^31,32^ Associations in tables were reported as odds ratios (ORs) alongside 95% compatibility (“confidence”) intervals (95% CIs).^33^ Throughout all analyses, age, and nicotine dependence were modeled using restricted cubic splines with three knots (placed at the minimum, median, and maximum for age, and scores of 1, 3, and 7 for dependence).^34,35^ This allows for flexible, non-linear relationships between these variables and the outcomes, increasing the precision and power of results while avoiding arbitrary categorization.^34^

### Indoor use for Vaping Versus Smoking

#### Exclusive Use

We reported the prevalence of indoor use among (i) people who exclusively smoke and (ii) people who exclusively vape. We compared the odds of indoor use between people who exclusively vape or exclusively smoke (reference category), before and after adjusting for covariates (age, sex, housing tenure, children or adults in the home, nicotine dependence) using logistic regression. To examine which places people vape or smoke indoors, we reported the percentage who use indoors in their homes, car, or other places.

#### Dual Use

For people who dual use cigarettes and e-cigarettes, we compared their odds of indoor use for vaping versus smoking (reference category) using hierarchical logistic regression, with random intercepts for each individual using the *lme4* package (unweighted, as it is a within-person comparison).^36^ We estimated the percentage of people who dual use that only vape indoors, only smoke indoors, both vape and smoke indoors, or neither vape nor smoke indoors.

### Differences by Demographics and Dependence

In people who use nicotine (ie, smoke and/or vape), we estimated how odds of indoor use differs by age, sex, housing tenure, presence of children or adults in the home, and nicotine dependence—before and after adjustment for all other covariates including whether someone exclusively smokes, exclusively vapes, or dual uses. We repeated the above analyses for (i) vaping indoors among people who exclusively vape—including smoking history as an additional covariate and (ii) smoking indoors among people who exclusively smoke—including vaping history as an additional covariate.

### Registration

The protocol for this study was registered prior to data analysis (https://osf.io/5cy37). There was one change from the protocol; we were not able to perform the supplementary regression discontinuity analysis across birth cohorts because age was only provided in 2- to 5-year bands.

## Results

Of the 8204 adults (aged ≥16) interviewed, 8157 (99.4%) reported both their smoking and vaping status, with 1537 (18.7%) reporting that they currently smoked or vaped. There were 1530 participants with complete data for all covariates, while seven (0.5%) had missing data (three for housing tenure, four for nicotine dependence). The analysis was restricted to the 1530 complete cases. Of these, 282 (18.4%) exclusively vaped, 1062 (69.4%) exclusively smoked, and 186 (12.2%) dual used both cigarettes and e-cigarettes. There were 752 (49.2%) males, 455 (29.7%) participants living with children, and 468 (30.6%) living in social housing. Mean age was 44.8 (standard deviation = 16.4), Demographic characteristics are provided in Table 1, stratified by smoking and vaping status.

**Table 1.** Sample Characteristics

Characteristic	Overall, N = 1530^1^	Exclusively vape, N = 282^1^	Exclusively smoke, N = 1062^1^	Dual use, N = 186^1^
Age	44.8 (16.4)	44.8 (14.7)	45.0 (16.9)	43.5 (16.2)
Sex (% male)	752 (49.2%)	146 (51.8%)	512 (48.2%)	94 (50.5%)
Housing tenure				
Owned	683 (44.6%)	156 (55.3%)	447 (42.1%)	80 (43.0%)
Private rent	379 (24.8%)	67 (23.8%)	268 (25.2%)	44 (23.7%)
Social rent	468 (30.6%)	59 (20.9%)	347 (32.7%)	62 (33.3%)
Living with others				
Alone	366 (23.9%)	42 (14.9%)	269 (25.3%)	55 (29.6%)
Only adults	709 (46.3%)	150 (53.2%)	476 (44.8%)	83 (44.6%)
Children	455 (29.7%)	90 (31.9%)	317 (29.8%)	48 (25.8%)
Nicotine dependence				
1 (Least dependent)	215 (14.1%)	42 (14.9%)	151 (14.2%)	22 (11.8%)
2	197 (12.9%)	29 (10.3%)	151 (14.2%)	17 (9.1%)
3	193 (12.6%)	33 (11.7%)	138 (13.0%)	22 (11.8%)
4	242 (15.8%)	53 (18.8%)	161 (15.2%)	28 (15.1%)
5	227 (14.8%)	39 (13.8%)	159 (15.0%)	29 (15.6%)
6	250 (16.3%)	47 (16.7%)	165 (15.5%)	38 (20.4%)
7 (Most dependent)	206 (13.5%)	39 (13.8%)	137 (12.9%)	30 (16.1%)

^1^Mean (SD) for continuous variables; *n* (%) for categorical variables. Unweighted data.

### Indoor Use for Vaping Versus Smoking

**Figure 1.**
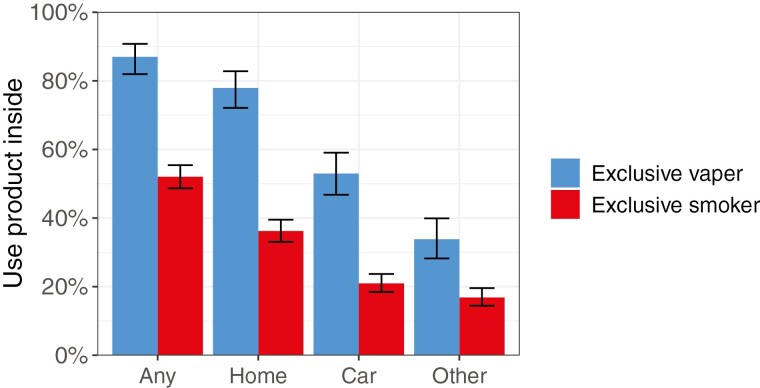
Prevalence of indoor use across different locations among people who exclusively vape (*N* = 282) or exclusively smoke (*N* = 1062). Estimates come from unadjusted logistic regressions accounting for survey design and weights. Error bars represent 95% CIs.

**Figure 2.**
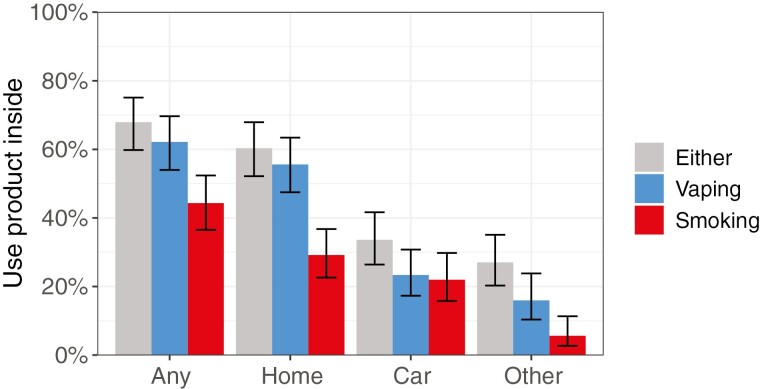
Prevalence of indoor vaping and smoking across different locations among people who dual use both cigarettes and e-cigarettes (*N* = 186). Estimates come from unadjusted logistic regressions accounting for survey design and weights. Error bars represent 95% CIs.

People who exclusively vaped were more likely than those who exclusively smoked to report having used their product indoors within the past seven days (87.0% vs. 52.0%; OR_un_ = 6.16, 95% CI = 4.09 to 9.28). This association strengthened after covariate adjustment (OR_adj_ = 9.34, 95% CI = 5.75 to 15.2). Similarly, people who dual used both e-cigarettes and cigarettes were more likely to report vaping than smoking indoors (62.1% vs. 44.3%; within-person OR = 3.76, 95% CI = 2.06 to 6.84; Figure 1); an estimated 23.6% of them only vaped indoors, 38.5% both smoked and vaped indoors, 5.8% only smoked indoors, and 32.1% reported no indoor use. The prevalence of indoor use was higher for vaping than smoking across all locations (indoor in the home, car, and other places), both in exclusive (Figure 1) and dual users (Figure 2), but the most common location for people to use either product indoors was the home.

### Differences by Demographics and Dependence

Table 2 shows differences in indoor use—that is, smoking and/or vaping indoors—by demographic factors and nicotine dependence, before and after covariate adjustment. Older adults were more likely to use their product indoors; for instance, the prevalence of indoor use was estimated at 87.9% in 80-year-olds compared with just 42.0% in 20-year-olds (OR_un_ = 10.07, 95% CI = 6.33 to 16.01). Indoor use was highest for people who lived alone (73.4%), followed by those only living with adults (62.2%; OR_un_ = 0.60, 95% CI = 0.43 to 0.83) and then by those living with children (46.1%; OR_un_ = 0.31, 95% CI = 0.22 to 0.44). Nicotine dependence was strongly associated with indoor use: 84.4% of the most dependent individuals used their product indoors compared with 34.6% of the least dependent users (OR_un_ = 10.22, 95% CI = 6.86 to 15.20).

**Table 2.** Correlates of Indoor Use in People Who Smoke and/or Vape (*N* = 1530)

	Use indoors^1^	OR_un_ (95% CI)^2^	OR_adj_ (95% CI)^2^
Age (years)			
20	42.0%	Ref	Ref
40	56.5%	1.79 (1.33 to 2.42)	1.23 (0.84 to 1.80)
60	73.7%	3.87 (2.75 to 5.43)	2.05 (1.30 to 3.23)
80	87.9%	10.07 (6.33 to 16.01)	4.35 (2.38 to 7.96)
Sex			
Female	57.7%	Ref	Ref
Male	62.1%	1.20 (0.95 to 1.51)	1.13 (0.85 to 1.51)
Housing tenure			
Owned	57.3%	Ref	Ref
Private rent	56.9%	0.98 (0.74 to 1.31)	1.33 (0.91 to 1.92)
Social rent	68.1%	1.59 (1.22 to 2.07)	1.62 (1.15 to 2.30)
Living with others			
Alone	73.4%	Ref	Ref
Only adults	62.2%	0.60 (0.43 to 0.83)	0.61 (0.42 to 0.90)
Children	46.1%	0.31 (0.22 to 0.44)	0.27 (0.18 to 0.42)
Nicotine dependence			
1 (Least dependent)	34.6%	Ref	Ref
2	42.8%	1.42 (1.17 to 1.72)	1.46 (1.20 to 1.77)
3	51.8%	2.04 (1.46 to 2.85)	2.15 (1.54 to 3.00)
4	61.2%	2.99 (2.03 to 4.39)	3.20 (2.18 to 4.71)
5	70.2%	4.45 (3.06 to 6.48)	4.85 (3.33 to 7.05)
6	78.0%	6.73 (4.69 to 9.64)	7.40 (5.16 to 10.60)
7 (Most dependent)	84.4%	10.22 (6.86 to 15.20)	11.33 (7.62 to 16.86)
Smoking/vaping status			
Exclusively smoke	52.0%	Ref	Ref
Dual use	67.9%	1.95 (1.34 to 2.84)	1.97 (1.28 to 3.03)
Exclusively vape	87.0%	6.16 (4.09 to 9.28)	8.83 (5.52 to 14.14)

^1^Percentage who self-reported indoor use within the past 7 days. Percentages are fitted values from unadjusted logistic regressions accounting for survey design and weights.

^2^OR_un_ = odds ratio, unadjusted for covariates; OR_adj_ = odds ratio, adjusted for age, sex, housing tenure, living with adults or children, nicotine dependence, and smoking/vaping status; 95% CI = 95% compatibility interval. Age and nicotine dependence were modeled non-linearly using restricted cubic splines.

Similar associations were found when looking specifically at predictors of vaping indoors among people who exclusively vape and smoking indoors among people who exclusively smoke (Table 3). An exception to this was housing tenure, which had different associations with smoking indoors and vaping indoors. Among people who smoke, the percentage who smoked indoors was higher in social housing (65.7%) than owner-occupied homes (44.9%; OR_un_ = 2.35, 95% CI = 1.72 to 3.20). However, among people who vape, the percentage of vaping indoors was similar or lower in social housing (81.4%) than in owner-occupied homes (91.4%; OR_un_ = 0.41, 95% CI = 0.16 to 1.10). Equivalent associations were found after covariate adjustment.

**Table 3.** Correlates of Indoor Use in People Who Exclusively Vape (*N* = 282) or Exclusively Smoke (*N* = 1062).

	In people who exclusively vape			In people who exclusively smoke		
	Vape indoors^1^ (%)	OR_un_ (95% CI)^2^	OR_adj_ (95% CI)^2^	Smoke indoors^1^ (%)	OR_un_ (95% CI)^2^	OR_adj_ (95% CI)^2^
Age (years)						
20	57.0	Ref	Ref	41.3	Ref	Ref
40	88.6	5.89 (2.89 to 11.99)	5.32 (3.69 to 7.69)	43.7	1.10 (0.77 to 1.58)	1.12 (0.79 to 1.60)
60	94.2	12.20 (5.90 to 25.25)	4.99 (3.23 to 7.70)	66.7	2.84 (1.93 to 4.20)	2.05 (1.34 to 3.13)
80	92.2	8.91 (3.08 to 25.79)	1.34 (0.75 to 2.41)	92.3	17.05 (10.62 to 27.36)	5.55 (3.05 to 10.08)
Sex						
Female	87.8	Ref	Ref	48.9	Ref	Ref
Male	86.3	0.87 (0.40 to 1.89)	0.60 (0.25 to 1.48)	54.7	1.26 (0.96 to 1.66)	1.34 (0.96 to 1.88)
Housing tenure						
Owned	91.4	Ref	Ref	44.9	Ref	Ref
Private rent	83.1	0.46 (0.19 to 1.14)	0.93 (0.28 to 3.06)	49.0	1.18 (0.83 to 1.67)	1.50 (0.97 to 2.32)
Social rent	81.4	0.41 (0.16 to 1.10)	0.46 (0.14 to 1.49)	65.7	2.35 (1.72 to 3.20)	2.28 (1.54 to 3.36)
Living with others						
Alone	97.6	Ref	Ref	69.4	Ref	Ref
Only adults	89.0	0.20 (0.02 to 1.62)	0.18 (0.02 to 1.70)	53.5	0.51 (0.35 to 0.74)	0.62 (0.40 to 0.96)
Children	79.0	0.09 (0.01 to 0.75)	0.06 (0.01 to 0.59)	35.0	0.24 (0.16 to 0.35)	0.28 (0.17 to 0.46)
Nicotine dependence						
1 (Least dependent)	74.9	Ref	Ref	24.2	Ref	Ref
2	80.0	1.34 (0.95 to 1.90)	1.00 (0.82 to 1.21)	33.1	1.55 (1.26 to 1.92)	1.49 (1.22 to 1.81)
3	84.4	1.81 (0.99 to 3.30)	1.08 (0.77 to 1.51)	43.4	2.41 (1.67 to 3.46)	2.22 (1.59 to 3.11)
4	88.1	2.48 (1.23 to 4.98)	1.35 (0.92 to 1.99)	54.1	3.70 (2.42 to 5.64)	3.36 (2.29 to 4.93)
5	91.1	3.42 (1.70 to 6.90)	1.91 (1.32 to 2.78)	64.3	5.65 (3.72 to 8.59)	5.12 (3.52 to 7.45)
6	93.4	4.76 (2.31 to 9.79)	2.94 (2.05 to 4.21)	73.3	8.60 (5.72 to 12.94)	7.86 (5.48 to 11.26)
7 (Most dependent)	95.2	6.64 (2.85 to 15.44)	4.69 (3.15 to 6.98)	80.7	13.07 (8.39 to 20.36)	12.09 (8.12 to 17.98)
Past regular smoking/vaping						
No	72.0	Ref	Ref	50.0	Ref	Ref
Yes	89.3	3.23 (1.27 to 8.23)	1.43 (0.44 to 4.65)	55.6	1.26 (0.94 to 1.67)	1.51 (1.06 to 2.14)

^1^Percentage who self-reported indoor use within the past 7 days. Percentages are fitted values from unadjusted logistic regressions accounting for survey design and weights.

^2^OR_un_ = odds ratio, unadjusted for covariates; OR_adj_ = odds ratio, adjusted for age, sex, housing tenure, living with adults or children, nicotine dependence, and past regular smoking (for exclusive vapers), or past regular vaping (for exclusive smokers); 95% CI = 95% compatibility interval. Age and nicotine dependence were modeled non-linearly using restricted cubic splines.

Among people who exclusively vape, the prevalence of indoors vaping was higher in those who had previously smoked regularly compared with those who had not (89.3% vs. 72.0%; OR_un_ = 3.23, 95% CI = 1.27 to 8.23), but the association weakened substantially after covariate adjustment (OR_adj_ = 1.43, 95% CI = 0.44 to 4.65). Conversely, among people who exclusively smoke, the prevalence of indoors smoking was slightly higher in those with versus without a history of regular vaping (55.6% vs. 50.0%, OR_un_ = 1.26, 95% CI = 0.94 to 1.67; OR_adj_ = 1.51, 95% CI = 1.06 to 2.14).

## Discussion

We found that adults in England were much more likely to vape indoors than smoke indoors. This was true both when comparing people who exclusively vape with those who exclusively smoke and when looking within people who dual use. Moreover, the preference for vaping over smoking indoors was found across different locations, including at home and in cars. People who were older, highly dependent on nicotine, and who lived alone were most prone to vape or smoke indoors. The association of housing tenure with indoors use differed for vaping and smoking; the majority of people vaping vaped indoors regardless of their housing status, whereas those living in social housing were more likely to smoke indoors than homeowners.

Previous data from the International Tobacco Control (ITC) Four Country Survey show that people in Canada, the US, England, and Australia are much more likely to have rules banning smoking than vaping indoors at home.^37^ Similar results were found in the U.S. Population Assessment of Tobacco and Health (PATH) study.^38^ Our results extend these findings by showing that these less restrictive rules towards vaping indoors in the home translate to differences in behavior. As mentioned in the introduction, there are several possible causes for why people feel more comfortable vaping than smoking indoors, including the less aversive odor and lower harmfulness. We did not have the data to explore the extent to which these factors explain differences in why indoor vaping is so prevalent, so future research should examine which factors are the most influential barriers and enablers for indoor vaping.

Certain characteristics were associated with whether someone would vape or smoke indoors. Over the past 20 years in England, parents who smoke increasingly do so outdoors to avoid harming their children.^5^ We found that having others in the home, especially children, may deter people from smoking or vaping indoors. However, concerns about harms are likely a less powerful deterrent for vaping indoors than smoking, as shown by high rates of vaping indoors—at 79%—among vapers who live with children. Indeed, these differing concerns might actually encourage some parents to vape indoors; a theme identified in interviews with women who had recently given birth was that they vape rather than smoke indoors to protect their child from secondhand smoke.^39^

Nicotine dependence was the most influential driver of both vaping and smoking indoors. This is likely because people who are heavily dependent on nicotine have stronger and more frequent urges to smoke and vape, making it inconvenient for them to go outdoors every time they wish to satisfy their cravings.^40^

Age was also strongly associated with indoor use, with the oldest adults being the most likely to both smoke and vape indoors. This may reflect older people having started smoking when it was common and normalized to smoke indoors (or more dependent on nicotine, if residual confounding remains after adjustment for time to first vape/smoke in the morning). Conversely, the youngest adults will have only started using nicotine after smoke-free laws were introduced that banned smoking indoors in restaurants, bars, and workplaces in England, and when smoking indoors became increasingly denormalized.^7,41^ The association with age may also be because of older individuals being more likely to have disabilities or limited mobility, making them less capable of going outdoors.

Our findings align with previous studies showing that people who smoke are drawn to e-cigarettes because they can use them indoor smoke-free places; data from the Smoking Toolkit Study in England show that three-quarters of adults who dual use e-cigarettes and cigarettes report vaping in places where smoking is not permitted.^42^ Even without laws against vaping indoors, many policy makers, businesses, and landlords have expanded or are considering expanding smoke-free policies to include vaping.^18,27,43^ Such decisions may come down to ethical considerations of how to balance the rights of bystanders to avoid the unpleasant aerosol and (albeit minimal) harm with the rights of people to choose where they vape. However, the effects of vaping indoors on behavior and thus public health should also be considered. Harms would arise if, for example, the sight of others vaping indoors encourages nicotine naïve youths to start vaping, renormalizes smoking indoors, or makes it more difficult for people to quit vaping (the higher prevalence of smoking indoors among those who did versus did not previously vape is consistent with renormalization). Conversely, benefits would arise if people vape indoors as an alternative to smoking indoors or as an aid to quitting smoking entirely. These potential harms and benefits are discussed in detail elsewhere.^44^

Our study had several strengths. While other studies have compared the adoption of vape- and smoke-free household rules,^37,38^ this is the first study to compare the prevalence of vaping with smoking indoors. It used a large and representative sample of the adults in England, guaranteeing the generalisability of findings. Importantly, we were able to compare the prevalence of vaping indoors with smoking indoors both (i) between participants who exclusively use each product and (ii) within participants who dual use. The within-person comparison provides some confidence that the preference for vaping over smoking indoors was not the result of confounding (ie, it was not caused by differences in the characteristics of people who choose to exclusively vape versus exclusively smoke).

There were limitations. Firstly, the survey relies on self-reported data, which may be subject to social desirability bias, especially for sensitive questions such as vaping or smoking indoors.^45^ However, previous studies have validated these self-reported measures against biological markers of secondhand smoke exposure.^46^ For instance, saliva cotinine levels are five-times higher in the children of parents’ who self-report smoking indoors in the home compared with those who report only smoking outdoors.^5^ Secondly, interviewers did not ask specifically about use indoors in bars, restaurants, or workplaces (although these are captured under “other places”). This meant we could not examine the prevalence of vaping indoors in public places where smoking is banned under 2007 smoke-free legislation. Thirdly, since our data were collected, harm perceptions of e-cigarettes relative to cigarettes have worsened substantially in England, so people may now be more wary of vaping indoors.^47,48^ Therefore, it is important to keep tracking the prevalence of indoor vaping and smoking. Fourthly, the measures of ever regular use were different for vaping and smoking (measured as past use more than once or twice for vaping but past daily use for smoking) so they cannot be directly compared. Finally, participants were only asked about indoor use within the past 7 days. It is likely that we underestimate the prevalence of indoor use for those who smoke/vape infrequently (eg, less than weekly).

To conclude, we found that adults in England have a clear preference for vaping over smoking indoors, with high nicotine dependence, living alone, and older age being the strongest correlates of indoor use of either product. As many places consider expanding smoke-free rules to also ban vaping indoors, there is a need to better understand the benefits, harms, and ethics of such policy changes.

## Supplementary material

Supplementary material is available at *Nicotine and Tobacco Research* online.

ntae094_suppl_Supplementary_Tables_S1-S2
