# Supplementary material for: Are People More Likely to Vape or Smoke Indoors? A Population Survey of Adults in England
Source: Nicotine Tob Res. 2024 Apr 18;26(10):1404–11. doi: 10.1093/ntr/ntae094 (PMC11417121; doi:10.1093/ntr/ntae094)
Supplement: ntae094_suppl_Supplementary_Tables_S1-S2 [file ntae094_suppl_supplementary_tables_s1-s2.docx]

**Supplementary Table 1: Prevalence of indoor use in people who exclusively vape (N=282) or exclusively smoke (N=1062).**

|  |  | **Use indoors** | | |
| --- | --- | --- | --- | --- |
| ***Location*** | ***Product*** | ***N*** | ***n^1^*** | ***% (95%CI)^1^*** |
| Any | Smoking | 1066 | 555 | 52.0 (48.6 to 55.4) |
| Any | Vaping | 283 | 246 | 87.0 (82.0 to 90.8) |
| Home | Smoking | 1066 | 386 | 36.2 (33.1 to 39.5) |
| Home | Vaping | 283 | 221 | 77.9 (72.1 to 82.8) |
| Car | Smoking | 1066 | 223 | 20.9 (18.4 to 23.7) |
| Car | Vaping | 283 | 150 | 53.0 (46.8 to 59.0) |
| Other | Smoking | 1066 | 180 | 16.8 (14.4 to 19.6) |
| Other | Vaping | 283 | 96 | 33.8 (28.2 to 39.9) |

^1^ Number (*n*) and percentage (%) who self-reported indoor use within the past seven days. Percentages are fitted values from unadjusted logistic regressions accounting for survey design and weights.

**Supplementary Table 2: Prevalence of indoor use in dual users of cigarettes and e-cigarettes (N=188).**

|  |  | **Use indoors** | | |
| --- | --- | --- | --- | --- |
| ***Location*** | ***Product*** | ***N*** | ***n^1^*** | ***% (95%CI)^1^*** |
| Any | Either | 188 | 128 | 67.9 (59.8 to 75.1) |
| Any | Vaping | 188 | 117 | 62.1 (54.0 to 69.7) |
| Any | Smoking | 188 | 83 | 44.3 (36.5 to 52.4) |
| Home | Either | 188 | 113 | 60.3 (52.2 to 67.9) |
| Home | Vaping | 188 | 105 | 55.6 (47.5 to 63.4) |
| Home | Smoking | 188 | 55 | 29.2 (22.6 to 36.8) |
| Car | Either | 188 | 63 | 33.6 (26.4 to 41.6) |
| Car | Vaping | 188 | 44 | 23.4 (17.3 to 30.8) |
| Car | Smoking | 188 | 41 | 22.0 (15.8 to 29.8) |
| Other | Either | 188 | 51 | 27.0 (20.3 to 35.1) |
| Other | Vaping | 188 | 30 | 16.0 (10.3 to 23.8) |
| Other | Smoking | 188 | 11 | 5.6 (2.7 to 11.3) |

^1^ Number (*n*) and percentage (%) who self-reported indoor use within the past seven days. Percentages are fitted values from unadjusted logistic regressions accounting for survey design and weights.
